# *Sorbuslushanensis*, a new species of Rosaceae from China

**DOI:** 10.3897/phytokeys.119.32148

**Published:** 2019-03-26

**Authors:** Jing Qiu, Yang Zhao, Qi Qi, Xin Chen

**Affiliations:** 1 Co-Innovation Center for Sustainable Forestry in Southern China, College of Biology and The Environment, Nanjing Forestry University, Nanjing 210037, Jiangsu China Nanjing Forestry University Nanjing China; 2 Sanjiang University, Nanjing 210012, Jiangsu, China Sanjiang University Nanjing China

**Keywords:** *
Sorbus
*, new species, taxonomy, China

## Abstract

*Sorbuslushanensis* Xin Chen & Jing Qiu, **sp. n.** (Rosaceae), a new simple-leaved species belonging to Sorbussubg.Ariasect.Alnifoliae, is described from Anhui and Jiangxi provinces in China. Illustrations, photographs of wild plants and a distribution map are presented. The new species is morphologically similar to *S.folgneri*, but can be distinguished easily by its abaxially greenish-grey tomentose leaves, scale-like stipules and glabrous styles.

## Introduction

Species of *Sorbus* are mainly distributed in the temperate areas of the Northern Hemisphere, with a centre of the highest diversity in East Asia, especially in China. The genus comprises about 100 (Lu and Spongberg 2003) to more than 250 (Phipps et al. 1990) species, 67 of which are native to China (Lu and Spongberg 2003). During fieldwork carried out in recent years for wild germplasm resources of the genus, a new simple-leaved species from Anhui and Jiangxi provinces in China was discovered and is described here.

## Materials and methods

The description was based on data and specimens collected in the field between 2015 and 2018 from Anhui and Jiangxi provinces. Geographical coordinates and elevations were determined using Holux m-241. Voucher specimens were deposited at the Herbarium of Nanjing Forestry University (**NF**).

## Taxonomic treatment

### Sorbus lushanensis

Taxon classificationPlantaeRosalesRosaceae

Xin Chen & Jing Qiu sp. n.

urn:lsid:ipni.org:names:77195769-1

Figures 1 , 2 , 3

#### Type.

China. Jiangxi: Lushan City, Lushan National Park, Xianren Cave, 993 m alt., 29°34'06.24"N, 115°57'42.84"E, 05 May 2018, *J. Qiu 1219* (holotype NF-2005029!; isotypes NF-2005027!, NF-2005028!, NF-2005030!, NF-2005031!, NF-2005032!, NF-2005033!, NF-2005034!, NF-2005035!)

#### Diagnosis.

*Sorbuslushanensis* is morphologically most similar to *S.folgneri* (C. K. Schneid.) Rehd., but differs by its leaf blade abaxially greenish-grey tomentose, stipules smaller, pedicels longer, petals larger and styles glabrous.

#### Description.

Tree up to 12 m tall, 14.6 cm in DBH; bole straight, bark grey to dark grey, smooth when young, with fissures, particularly at the base of trunk when mature. Branchlets greyish-brown, sparsely tomentose when young, glabrous or glabrescent at maturity, with pale brown to ochraceous lenticels. Buds turbinate or ovoid, pointed; scales reddish-brown, with white pubescent along margins. Leaves simple; stipules scale-like, 1–1.5 mm long, early deciduous; petiole (9–)13–16 (–19) mm long, greenish-grey tomentose; blades elliptic to broadly ovate, 5.9–9.2 cm long, (3–)4–5.6 cm wide, base cuneate to subcordate, apex acute to shortly acuminate, margin serrate to double serrate, densely greenish-grey tomentose abaxially, sparsely white tomentulose when young, gradually glabrous or glabrescent adaxially; venation craspedodromous, secondary veins 11–16 pairs, slightly impressed adaxially, raised abaxially. Inflorescence a compound corymb, terminal or axillary in the terminal 1–3 leaves, loosely 11–17(–26)-flowered; peduncles 3.9–6 cm long, pedicels 9.4–18.6 mm long, both sparsely white vilous. Flowers 12.5–14.2 mm in diam.; hypanthium campanulate, sparsely white vilous abaxially; sepals triangular-ovate, apex acute, 2.3–3.1 mm long, 2–2.9 mm wide, white vilous on both sides; petals white, broadly ovate or sub-rounded, apex obtuse, 5.9–7.1 mm long, 4–6.1 mm wide, glabrous, with a short claw at base. Stamens 17–20, 4.9–6.2 mm long, filaments whitish, anthers cream white to slightly yellow. Ovary 2-loculed, white tomentose apically. Styles 2, 3.1–4.6 mm long, connate to 1/3–1/2 of their length, glabrous. Infructescence glabrous, with numerous lenticels. Fruit orange-red, oblong to ovoid-oblong, 7.8–11.3 mm long, 4.2–7 mm in diam., 2-loculed, sparsely lenticellate, with an annular scar of the deciduous sepals and white tomentose inside it apically. Seeds brown, 5.46–6.48 mm long, 2.88–3.62 mm in diam., 2.04–2.72 mm thick.

**Figure 1.**
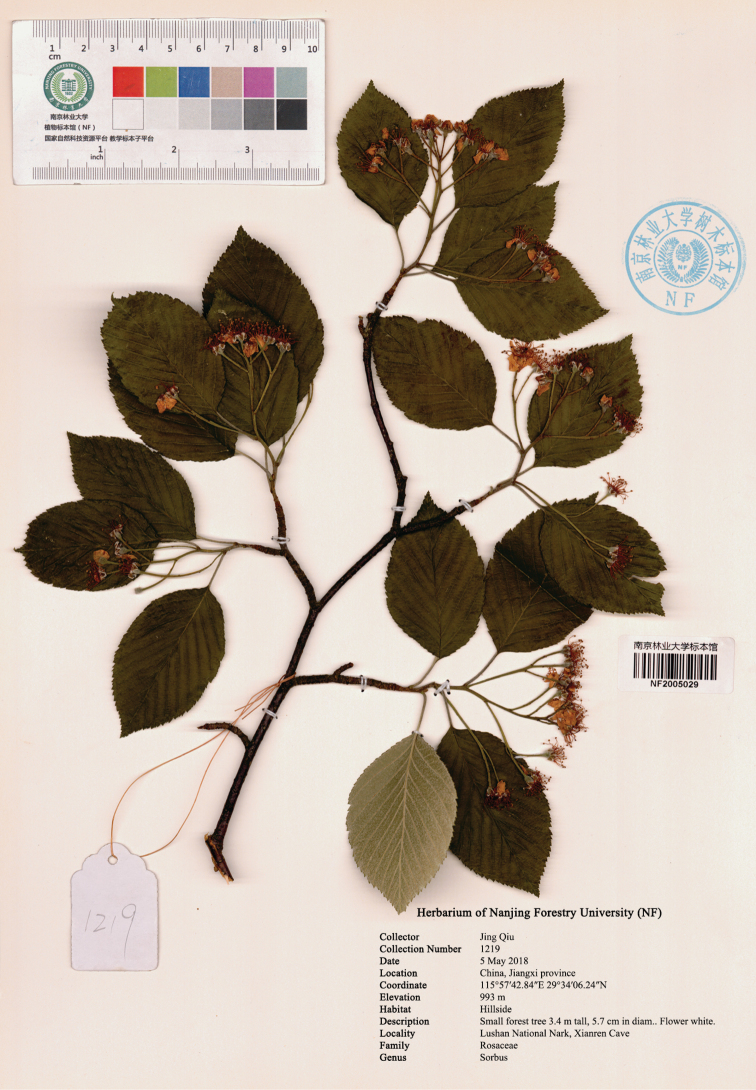
Holotype of *Sorbuslushanensis* sp. n. Scanned by Xiaochen Zhang.

**Figure 2.**
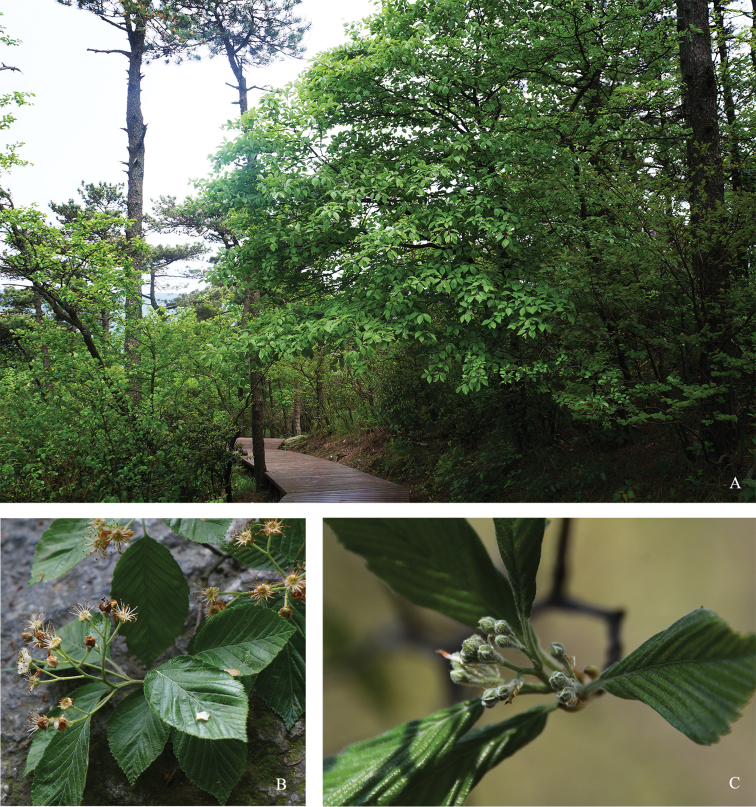
*Sorbuslushanensis* sp. n. **A** habit (A plant at Wulao Peak, Lushan National Park, Jiangxi province) **B** flowering branch and leaves (from the plant of type specimen) **C** young inflorescence (from the same plant as habit).

**Figure 3.**
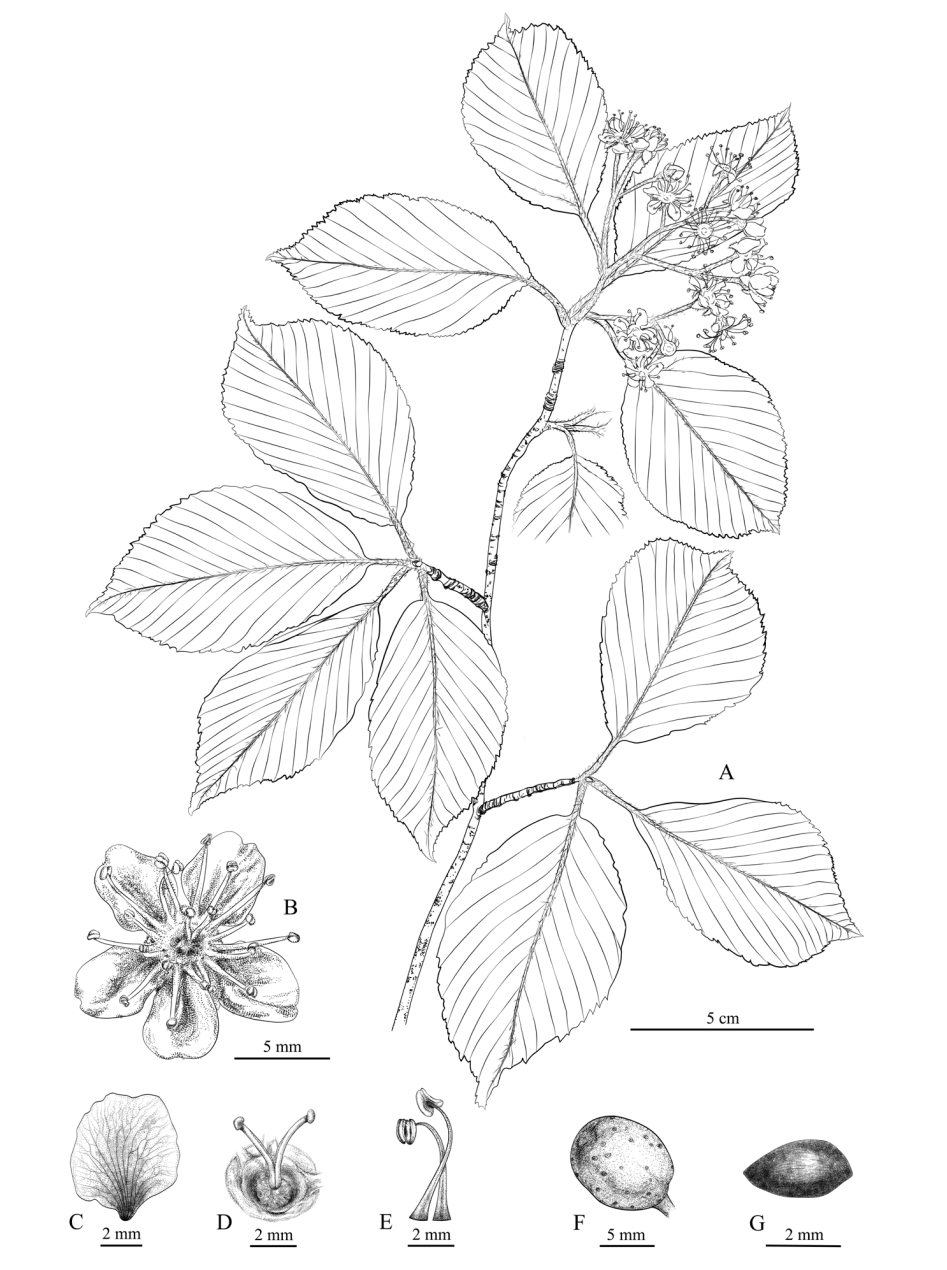
*Sorbuslushanensis* sp. n. **A** flower branch and leaves **B** flower **C** petal **D** styles **E** stamens **F** fruit **G** seed. Drawn by Yuxuan Bao.

#### Phenology.

Flowering from late April to early May, fruiting from September to October.

#### Etymology.

The name “*lushanensis*” refers to the type locality, Lushan Mountain, Jiangxi Province, China.

#### Vernacular name.

庐山花楸 (lu shan hua qiu).

#### Distribution, ecology and conservation status.

**Figure 4.**
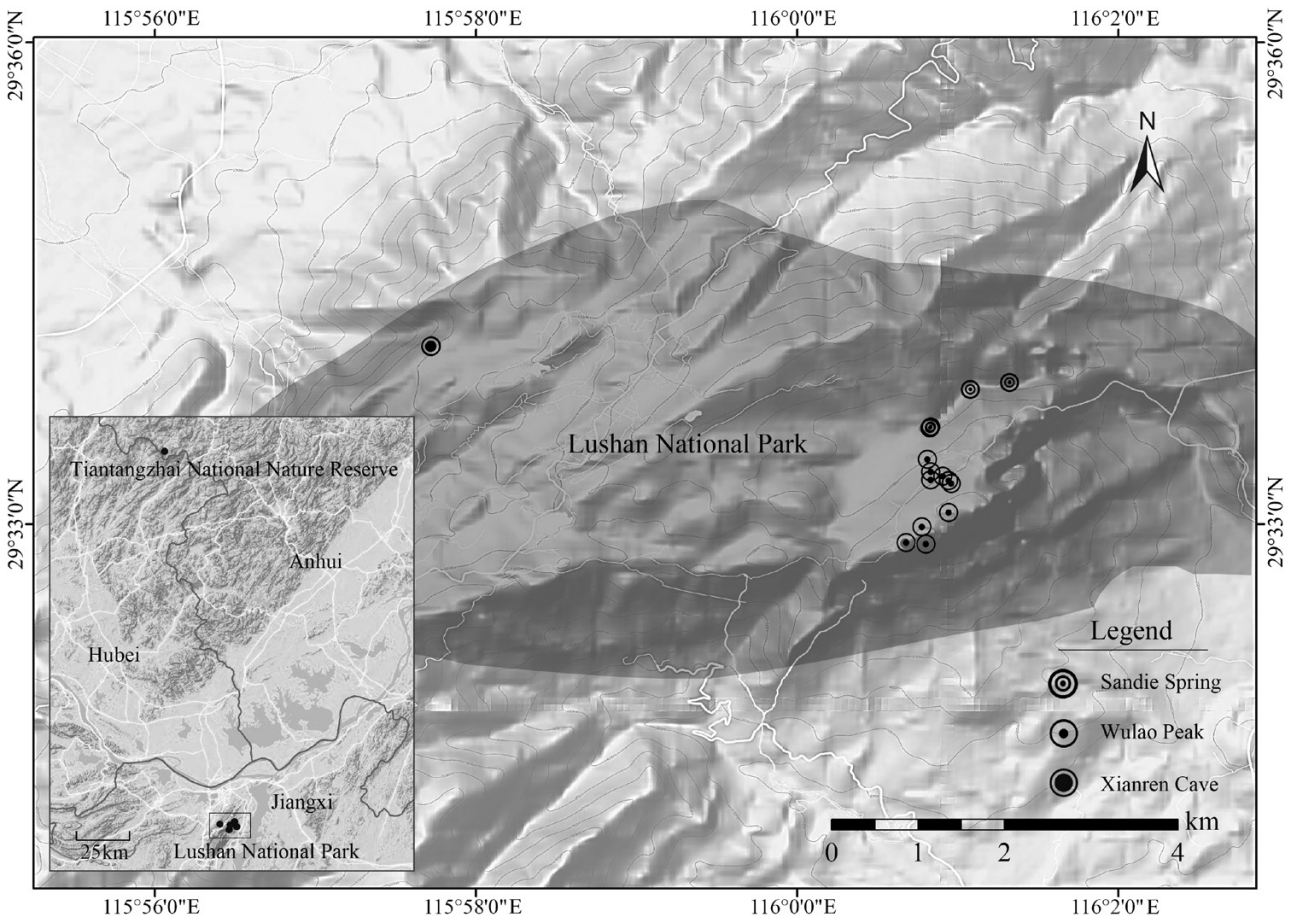
Geographic distribution of *Sorbuslushanensis* sp. n. Drawn by Junsheng Shu.

*Sorbuslushanensis* is presently known only from Anhui and Jiangxi provinces (Figure 4). It was observed growing in broad-leaved and mixed conifer broad-leaved forests at altitudes between 853 m and 1354 m, together with *S.alnifolia* (Siebold & Zucc.) K. Koch and *S.folgneri* from the same genus and *Pinustaiwanensis* Hayata, Carpinuscordatavar.chinensis Franch., Cornuskousasubsp.chinensis (Osborn) Q. Y. Xiang, *Cyclobalanopsisglauca* (Thunb.) Oerst., *Fraxinuschinensis* Roxb., *Litseaelongata* (Nees ex Wall.) Benth. & Hook. f., *Prunusserrulata* Lindl. etc. Its natural habitat is at core Lushan National Park and Tiantangzhai National Nature Reserve, which are perfectly protected. No threats were identified though only about 26 individuals were found along the collection routes. The diameter of individuals ranged from 1.5 to 14.6 cm, denoting the species regenerated well naturally. Adequate data on its distribution and population status need to be further collected for we investigated just along the tourist route without entering the inner forest. At present, we assign the conservation status of *S.lushanensis* as “Data Deficient (DD)” following the IUCN Red List Criteria and Categories (IUCN 2017).

#### Additional specimens examined.

China. Anhui: Lu’an City, Tiantangzhai National Nature Reserve, 853 m alt., 31°09'00.84"N, 115°46'07.39"E, 8 May 2015, *W. Du* & *F. Wang 0125* (NF). Jiangxi: Lushan City, Lushan National Park, Wulao Peak, 1354 m alt., 29°32'52.23"N, 116°00'47.78"E, 14 May 2016, *X. Chen, X. X. Fu* & *Q. L. Liu 0285* (NF); Wulao Peak, 1334 m alt., 29°33'04.00"N, 116°00'56.31"E, 14 May 2016, *X. Chen, X. X. Fu* & *Q. L. Liu 0289* (NF); Wulao Peak, 1246 m alt., 29°33'14.84"N, 116°00'57.31"E, 14 May 2016, *X. Chen, X. X. Fu* & *Q. L. Liu 0298* (NF); Wulao Peak, 1210 m alt., 29°33'17.49"N, 116°00'54.14"E, 14 May 2016, *X. Chen, X. X. Fu* & *Q. L. Liu 0302* (NF); Wulao Peak, 982 m alt., 29°33'16.12"N, 116°00'49.56"E, 14 May 2016, *X. Chen, X. X. Fu* & *Q. L. Liu 031*6 (NF); Wulao Peak, 1234 m alt., 29°33'15.88"N, 116°00'56.26"E, 25 April 2015, *X. Chen* & *W. Du 0069* (NF); Wulao Peak, 1089 m alt., 29°33'23.85"N, 116°00'48.32"E, 10 May 2015, *W. Du* & *F .wang 0085* (NF); Wulao Peak, 1306 m alt., 29°32'52.74"N, 116°00'40.44"E, 15 October 2015, *X. Chen, W. Q. Liu*; *M. W. Geng 0154* (NF); Wulao Peak, 1101 m alt., 29°33'19.14"N, 116°00'49.64"E, 15 October 2015, *X. chen, W. Q. Liu* & *M. W. Geng 0155* (NF); Wulao Peak, 1310 m alt., 29°32'58.59"N, 116°00'46.29"E, 15 October 2015, *X. chen, W. Q. Liu* & *M. W. Geng 0157* (NF); Sandie Spring, 1021 m alt., 29°33'36.10"N, 116°00'49.59"E, 25 April 2015, *X. Chen* & *W. Du 0063* (NF); Sandie Spring, 927 m alt., 29°33'49.98"N, 116°00'56.31"E, 25 April 2015, *X. chen* & *W. Du 0064* (NF); Sandie Spring, 897 m alt., 29°33'52.56"N, 116°01'19.04"E, 15 October 2015, *X. Chen, W. Q. Liu* & *M. W. Geng 0156* (NF); Sandie Spring, 983 m alt., 29°33'35.62"N, 116°00'49.31"E, 15 October 2015, *X. Chen, W. Q. Liu* & *M. W. Geng 0158* (NF); Xianren Cave, 987 m alt., 29°34'06.06"N, 115°57'42.78"E, 14 May 2016, *X. Chen*, *X. X. Fu* & *Q. L. Liu 0267* (NF).

#### Discussion.

**Figure 5.**
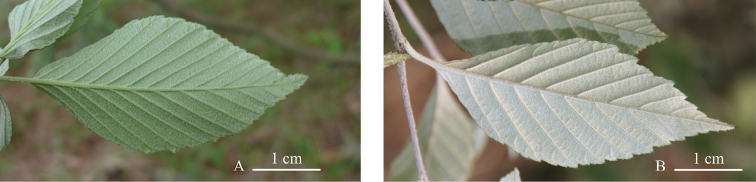
Leaf abaxial surface of **A***Sorbuslushanensis* and **B***S.folgneri*, show the similar of leaf form and the differences of hairs.

Morphological characters, such as sepals persistent or deciduous, leaves glabrous or with white or brown hair, venation craspedodromous or camptodromous, styles free or connate, are of taxonomic significance and useful in classification and delimitation of simple-leaved *Sorbus* taxa (Hedlund 1901, Yu and Lu 1974, Gabrielian 1978, Lu and Spongberg 2003, Aldasoro et al. 2004). In the latest revision of simple-leaved species of *Sorbus* (Aldasoro et al. 2004), species in China are assigned to one subgenus and five sections. The new species is morphologically a member of S.subg.AriaPersoonsect.Alnifoliae (Yu) Aldasoro et al., for it shares the common characters of this section, such as craspedodromous venation, spreading and white petals, coalescent styles and red pomes. Sorbussect.Alnifoliae contains five species: *S.alnifolia*, *S.japonica* (Decne.) Hedl., *S.zahlbruckneri* C. K. Schneid., *S.yuana* Spongberg and *S.folgneri*, mainly distributed in China, Japan and Korea (Aldasoro et al. 2004). The new species is morphologically similar to *S.folgneri* in leaf form (Figure 5) and the difference between the two species is summarised in Table 1.

**Table 1.** Comparison of main features separating *S.lushanensis* and *S.folgneri*.

	*** S. lushanensis ***	*** S. folgneri ***
Leaf blade	denssely greenish-grey-tomentose abaxially	densely white-tomentose abaxially
Stipule	scale-like, 1–1.5 mm long	lanceolate, 3–4.5 mm long
Petiole	sparsely greenish-grey-tomentose	densely white-tomentose
Inflorescence	sparsely white vilous	densely white tomentose
Pedicel	9.4–18.6 mm long	5–8 mm long
Petal	5.9–7.1 mm× 4–6.1 mm	5–5.8 mm× 2.5–4.2 mm
Style	glabrous	tomentose basally

*Sorbuslushanensis* and the two sympatric species, *S.alnifolia* and *S.folgneri* are all diploid (2n=2x=34) sexual species (Chen et al. unpubl. data). Our preliminary molecular work, based on two chloroplast gene fragments, atpB-rbcL and trnL and four nuclear gene fragments, GBSSI, PGIP, PPO and WD, resolved that *S.lushanensis* formed as sister to S.alnifoliavar.angulata S. B. Liang and they two together were resolved as sister to *S.folgneri*, suggesting that *S.lushanensis* is not most closely related to *S.folgneri* (Chen et al. unpubl. data). The new species can be distinguished from the other five species by its abaxially greenish-grey tomentose laminae.

## Key to the species of Sorbussect.Alnifoliae

**Table:** 

1	Leaves tomentose abaxially	**2**
–	Leaves glabrous or sparsely hairy abaxially	**4**
2	Leaves orbicular-ovate or suborbicular, margins lobed	*** S. japonica ***
–	Leaves elliptic to broadly ovate, margins serrate to double-serrate	**3**
3	Leaves densely white tomentose abaxially	*** S. folgneri ***
–	Leaves densely greenish-grey tomentose abaxially	*** S. lushanensis ***
4	Fruits with a small annular scar apically, sepals deciduous	*** S. alnifolia ***
–	Fruits with persistent sepals apically	**5**
5	Leaves elliptic to broadly ovate; fruits much larger, 10–16 × 6–13 mm	*** S. yuana ***
–	Leaves lanceolate; fruits much smaller, 6–10 × 4–7 mm	*** S. zahlbruckneri ***

## Supplementary Material

XML Treatment for Sorbus lushanensis
